# The impact of thermal cycling on *Staphylococcus aureus* biofilm growth on stainless steel and titanium orthopaedic plates

**DOI:** 10.1186/s12891-018-2199-z

**Published:** 2018-07-27

**Authors:** Margarete K. Akens, Claudia Chien, Ryan N. Katchky, Hans J. Kreder, Joel Finkelstein, Cari M. Whyne

**Affiliations:** 1 Techna Institute, University Health Network, 101 College Street, Rm 15-311, Toronto, ON M5J 2S2 Canada; 2 Department of Medical Biophysics, University of Toronto, Toronto, ON Canada; 3 Department of Surgery, University of Toronto, Toronto, ON Canada; 4 Sunnybrook Research Institute, 2075 Bayview Ave, Toronto, ON M4N 3M5 Canada

**Keywords:** Implant infection, Biofilm, Thermal cycling, Stainless steel plates, Titanium plates

## Abstract

**Background:**

Orthopaedic implant infections are difficult to eradicate because bacteria adhering to implant surfaces inhibit the ability of the immune system and antibiotics to combat these infections. Thermal cycling is a temperature modulation process that improves performance and longevity of materials through molecular structural reorientation, thereby increasing surface uniformity. Thermal cycling may change material surface properties that reduce the ability for bacteria to adhere to the surface of orthopaedic implants. This study aims to determine whether thermal cycling of orthopaedic implants can reduce bacterial growth.

**Methods:**

In a randomized, blinded in-vitro study, titanium and stainless steel plates treated with thermal cycling were compared to controls. Twenty-seven treated and twenty-seven untreated plates were covered with 10 ml tryptic soy broth containing ~ 10^5^ colony forming units (CFU)/ml of bioluminescent *Staphylococcus aureus* (*S. aureus)*Xen29 and incubated at 37 °C for 14d. Quantity and viability of bacteria were characterized using bioluminescence imaging, live/dead staining and determination of CFUs.

**Results:**

Significantly fewer CFUs grow on treated stainless steel plates compared to controls (*p* = 0.0088). Similar findings were seen in titanium plates (*p* = 0.0048) following removal of an outlier. No differences were evident in live/dead staining using confocal microscopy, or in metabolic activity determined using bioluminescence imaging (stainless steel plates: *p* = 0.70; titanium plates: *p* = 0.26).

**Conclusion:**

This study shows a reduction in CFUs formation on thermal cycled plates in-vitro. Further in-vivo studies are necessary to investigate the influence of thermal cycling on bacterial adhesion during bone healing. Thermal cycling has demonstrated improved wear and strength, with reductions in fatigue and load to failure. The added ability to reduce bacterial adhesions demonstrates another potential benefit of thermal cycling in orthopaedics, representing an opportunity to reduce complications following fracture fixation or arthroplasty.

## Background

Orthopaedic implant infection represents a devastating complication following surgical procedures, including fracture fixation, and reconstructive procedures [1–4]. Recently, there has been increased attention to the interactions between bacteria and the surface of orthopaedic implants [5]. Implant related infections occur due to bacterial adhesion onto implant surfaces, with subsequent biofilm formation. Biofilm producing bacteria can be highly resistant to antibiotic therapy and the native immune system, and as such, inhibiting bacterial adhesion to implants is crucial in preventing infections [6].

Biofilm formation on surfaces depends on biological, chemical and physical factors. The bacteria mainly influence biological factors and the material mainly determines chemical and physical factors. Chemical material composition, surface roughness and surface energy play an important role with regard to the interaction between the bacterium and the material surface [7]. Different weak physical forces such as van der Waal forces play a role in the adhesion of bacteria to the implant surface [8]. *Staphylococcus aureus* is weakly negatively charged, and therefore attracted to positively charged areas on the surface of orthopaedic implants [8]. Additionally, increasing surface roughness produces increased surface area and depressions within the implant surface, providing favourable sites for biofilm formation [9].

Thermal cycling is a proprietary patented (patent No. US 7,464,593 B1) temperature modulation process developed to improve the performance, strength and longevity of a variety of materials, including stainless steel, cast iron, aluminum, titanium, brass and copper [10, 11]. Described as ‘advanced cryogenics’, thermal cycling has been applied primarily to metals as an efficient, clean, non-polluting process that is currently utilized by a number of industries where improved performance is desired, including automotive, aerospace, manufacturing, electronics, construction tools, consumer products and sports equipment [12]. Thermal cycling has been used with great success in these industries, and has been shown to significantly improve performance and wear life of mechanical components, frequently about a five-fold increase in product life. Recently, the thermal cycling process has been demonstrated to be effective in significantly increasing the wear life and performance of surgical instruments [13].

During the thermal cycling process, materials are cooled and subsequently heated until they undergo molecular reorganization. This reorganization ‘tightens’ or optimizes the particulate structure of the material throughout, relieving stresses, and making it more dense and uniform, thereby minimizing flaws or imperfections. The reorganized structure also enhances the energy conductivity and heat distribution characteristics of the material. Thermal cycling does not alter the chemical composition of the material and therefore does not affect biocompatibility [11].

Based on this principle, the thermal cycling process may allow orthopaedic implants to have smoother, uniform surfaces. The surface changes and surface stress reduction may lead to decreased van der Waals forces and other physical forces without impacting the material composition [12]. If these molecular surface changes secondary to thermal cycling contribute to a reduction in bacterial adhesion, it could have the potential to reduce the incidence of orthopaedic implant infections. The purpose of this study was to evaluate the effect of thermal cycling on bacterial adhesion to orthopaedic stainless steel and titanium implants.

## Methods

### Materials

Commercially available stainless steel and titanium plates (Synthes, Mississauga, Canada) were double packaged, sterilized using an autoclave and randomly allocated to thermal cycled and control groups. The experimenters were blinded to the groups. The experiment using the stainless steel plates (2.0 mm straight plate, 4 holes; 23 mm, article # 243.14) with 6 plates/group was repeated 3 times (*n* = 18/group); the experiment using the titanium plates (2.0 mm Titanium zygomatic plates (dynamic compression plate (DCP), 4 holes. Article # 443.44) was repeated twice (*n* = 9/group).

### Thermal cycling

Plates allocated to the thermal cycled group were provided to Thermal Technology Services Limited (Vaughan, Canada) and underwent the thermal cycling procedure (patent No. US 7,464,593 B1).

Thermal cycling is a metallurgic treatment, which includes a ramp down, cold soak and ramp up operational procedures. Due to the mass and density of the plates, only one cycle was required. The temperature was ramped down over 2 h to - 148.9 °C, the plates exposed to the liquid nitrogen vapor at that temperature for 2 h and over a period of 16 h the temperature was increased to 20 °C. Visible and tactile inspection of the sterilisation pouches after thermal cycling did not show any alteration of the sterile packaging nor the macroscopic appearance of the plates. However, if thermal cycling of packaged implants is desired further sterility tests will be required prior to surgical use.

### Biofilm formation

A genetically engineered bioluminescent strain of *Staphylococcus aureus* - Xen29 - (PerkinElmer, Woodbridge, Canada) able to produce a biofilm was used in all experiments. It possesses a stable copy of the *Photorhabdus luminescens lux* operon allowing the bacteria to be bioluminescent without the addition of luciferin. *S. aureus* Xen29 cultures – in exponential phase growth - were diluted in tryptic soy broth with 0.25% glucose (TSBG broth) to a concentration of 10^5^ colony forming units (CFU)/ml. The implants were incubated on an orbital shaker with 10 ml of the diluted TSBG broth in 6-well plates at 37 °C and 70 rpm for 2 weeks. TSBG broth was changed every 24–48 h for 14 days, to enable the biofilm of *S. aureus* to reach maturity [14]. Biofilm formation was confirmed using bioluminescence and confocal imaging [15].

### Metabolic activity

Adequate oxygen level within a bacterium allow for the oxidation of reduced flavin mononucleotide (FMNH_2_) causing a *lux*-expressing bacterium to be constantly bioluminescent. The link of bioluminescence to bacterial metabolic activity has been used to test antimicrobial activity in real time [16]. Prior to imaging, the plates were rinsed with phosphate buffered saline (PBS) without CaCl_2_ and MgCl_2_ and placed in new 6-well plates with fresh TSBG. Bioluminescent images were obtained on day 3, 7, 10 and 14 using the IVIS Spectrum (Perkin Elmer, Waltham, MA, USA).

### Confocal imaging

Viability and growth pattern of the bacteria and biofilm were assessed using live/dead staining (BacLight™, Thermo Fisher Scientific, Mississauga, Canada). The plates were imaged using a Lab-Tek™ II2 chambered coverglass slide containing 1.5 ml fresh TSBG and 1.5 ml of 1× LIVE/DEAD® BacLight™. Imaging was performed using a Zeiss LSM700 confocal microscope (Carl Zeiss AG, Oberkochen, Germany) using the 488 nm and 555 nm laser.

### Colony forming units

The number of viable *S. aureus* Xen 29 colony forming units (CFU) contained in the biofilm were determined using traditional dilution methods. At the end of the experiment on day 14, each plate was placed in an Eppendorf tube containing 1 ml PBS and sonicated for 10 min in water at room temperature. The biofilm dilution was homogenized using micropipette aspiration. Serial dilutions up to 10^− 9^ were plated on lysogeny broth (LB) agar plates without antibiotics. After 24 h incubation at 37 °C CFU’s were counted and total numbers calculated using the dilution factor.

### Profilometry

The surface roughness of 12 stainless steel plates (*n* = 6 control; n = 6 thermal cycled) was determined using profilometry (Fort Bruce Testing Inc., London, Canada). A stylus Tencor P-10 surface profiler was used to acquire the surface profiles applying a stylus force of 3 mg. At a scan rate of 50 μm/s each plate was scanned over a length of 1250 μm. The results are presented as average surface roughness (Ra) in μm.

### Statistical analyses

Data analysis was completed using GraphPad Software, (GraphPad Software, Inc., La Jolla, CA, USA). Using a Mann-Whitney U test a *p*-value of 0.05 was considered statistically significant.

## Results

**Fig. 1:**
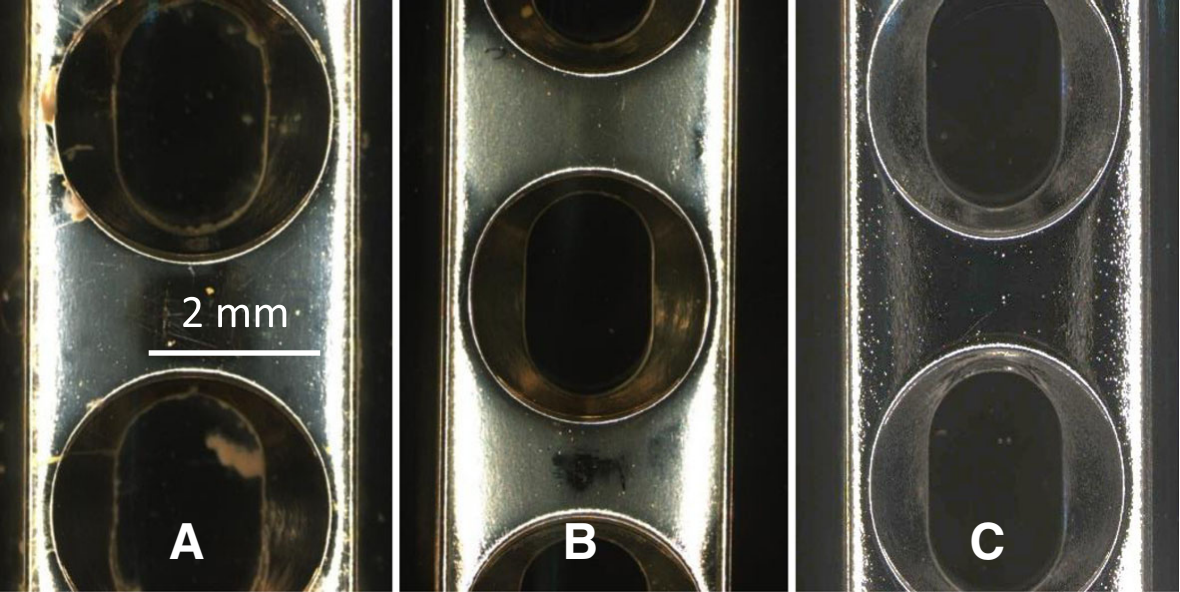
*S. aureus* Xen29 has been exposed to stainless steel plates for 14d. The control plate (**a**) shows a higher amount of biofilm accumulation compared to the thermal cycled plate (**b**). A new plate prior to bacteria exposure (**c**)

**Fig. 2:**
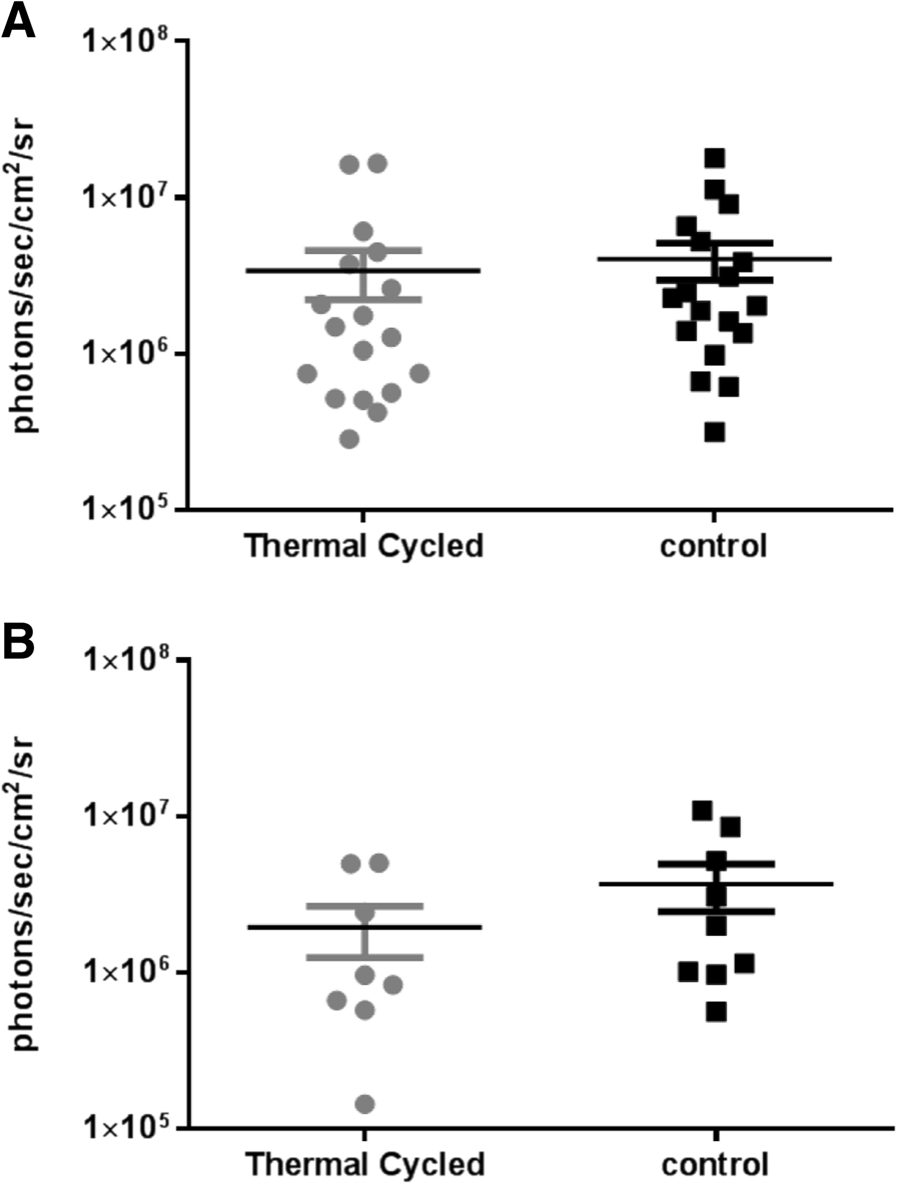
The graphs shows bioluminescent signals in photons/second/cm^2^ /steradian of *S. aureus* Xen29 on the control and thermal cycled stainless steel (**a**) and titanium plates (**b**) at day 14 in culture

Biofilm formation was achieved on all plates (Fig. 1). Nevertheless, the bioluminescence data showed no significant difference, indicating that there was no change in the metabolic activity of the bacteria growing on thermal cycled plates compared to untreated plates in both groups (stainless steel: *p* = 0.70; titanium: *p* = 0.25) (Fig. 2).

Two plates per group per repeat were used to perform live/dead staining. Although different growth patterns could be detected (Fig. 1), no differences were evident with respect to live/dead staining under confocal microscopy between the control and thermal cycled group. Thermal cycling did not have an effect on the viability of the bacteria.

**Fig. 3:**
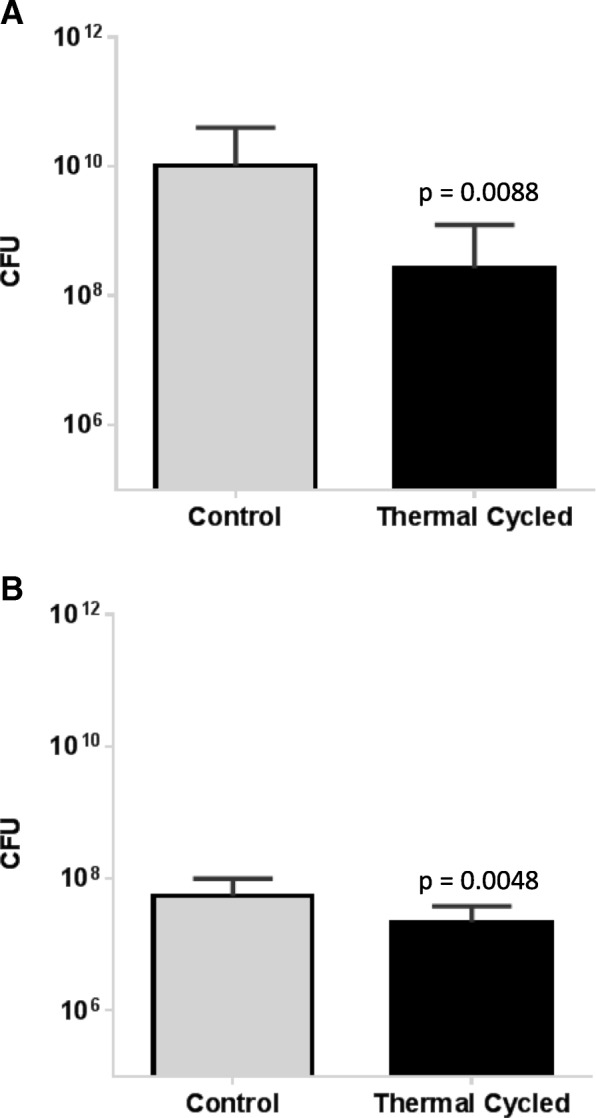
Growth of *S. aureus* Xen29 was assessed on control and thermal cycled stainless steel (**a**) and titanium plates (**b**) quantifying CFUs. Data represent the mean of three (stainless steel) or two (titanium plates) repeats, respectively. Error bars show standard deviation

However, significantly more bacteria capable of forming colonies grew on the control stainless steel plates compared to the plates that underwent thermal cycling (10^10^ vs. 10^8.5^, *p* = 0.0088). Similar findings were seen in the titanium plates (10^7.7^ vs. 10^7.3^, *p* = 0.0048) with the removal of outliers deriving from one single plate, likely caused by a contamination (Fig. 3).

Thermal cycling has been shown to influence surface roughness and therefore this parameter was measured in a subset of the stainless steel plates. The surface roughness measurement values obtained from the 12 stainless steel plates ranged from 0.07 to 0.12 μm (0.10 ± 0.01 μm). The surface roughness measurements (μm) were correlated to the CFU of the bacteria growing on the plates using Pearson correlation. No difference was found with respect to surface roughness between thermal cycled and control plates. Furthermore, there was no correlation of roughness with CFU formation (Spearman *r* = 0.1296). Due to this result, the surface roughness measurement were not repeated on the titanium plates.

## Discussion

The results of this study demonstrate that thermal cycling of stainless steel and titanium surgical plates yields a reduction of bacterial colony forming units. This suggests that thermal cycling may have the potential to decrease biofilm formation on orthopaedic implants.

The formation of colony forming units was selected as an appropriate primary outcome measure, as it represents the number of bacteria able to survive, cause an infection, and perpetuate new biofilm formation [17]. The reductions in CFUs demonstrated in this study suggest that thermal cycling does inhibit bacterial adherence and biofilm formation based on the surface changes of the material undergoing thermal cycling. The similarity between the two groups in live/dead staining under confocal microscopy and metabolic activity with bioluminescence is consistent with the proposed mechanism of thermal cycling – it does not have a bactericidal effect, but simply alters the surface properties of the implant, thereby reducing bacterial adherence.

The concept of ‘the race for the surface’, in which bacterial cells and host cells compete to colonize a newly introduced implant, is described in the literature [18]. Several different surface modifications have been proposed in efforts to create infection-resistant surfaces, including the development of anti-adhesive polymers [19], hydrophobic surfaces and nano-scale surface roughness [20]. However, there is concern that these measures may interfere with osteointegration [21]. Thermal cycling would likely have less of an impact on osteointegration than the aforementioned methods, given that the composition of the implant is not inherently altered. The process causes reduction in surface stress, which influences the physical forces followed by decreased adhesion of the bacteria [12]. With regard to surface roughness, all the values are below the 0.2 μm roughness threshold suggested for bacterial plaque retention [22]. The threshold of 0.2 μm was established on the theory of bacterial adhesion and retention based on the physio-chemically interaction of the bacterium and the surface. Decreasing the value below 0.2 μm did not show a difference in bacterial adhesion compared to higher values [22].

Both titanium and stainless steel plates were assessed as part of this study, with reductions in CFUs. These two materials comprise the vast majority of plates used for orthopaedic applications [23].

Based on previous studies, thermal cycling has been demonstrated to have significant positive effects on wear properties, corrosion and strength of metallic devices [11, 12]. Furthermore, the previously demonstrated reductions in metallic fatigue strength and maximum load to failure would be greatly beneficial for orthopaedic implants [24]. Thermal cycling has been shown to be relevant for biomedical and orthopaedic applications, through its ability to improve strength and reduce wear of surgical instruments [13]. Combining these previously observed benefits of thermal cycling with the ability to reduce bacterial adhesion could potentially have benefits in reducing complications associated with orthopaedic implants.

This study represents an important ‘proof of concept’, indicating the potential benefits of thermal cycling in reducing bacterial adherence to orthopaedic implants. This study included both titanium and stainless steel implants, indicating that the thermal cycling process is applicable to metallic alloys used in orthopaedic surgeries. A rigorous protocol was developed for both inoculation of implants and quantification of bacterial adhesion, in keeping with accepted standards [25, 26]. Reproducibility was demonstrated throughout experimental trials by CFU results with low variance. However, the presence of outliers skewed the data. All outlying results were derived from a single titanium plate, suggesting that this plate may have been affected by unappreciated confounding factor, producing significantly higher bacterial adhesion than all other thermal cycled plates. This outlier was removed from the analysis since it fell outside of the 1.5 times interquartile range (IQR), in keeping with accepted statistical analysis standards [27]. Once this outlier was removed from the results, the thermally cycled titanium plates demonstrated a statistically significant reduction in CFUs, similar to the results obtained for stainless steel plates. Future in-vitro studies could evaluate CFUs at additional time-points and the potential impact of thermal cycling on osteoblast adherence and growth at the bone implant interface.

The thermal cycling led roughly to a 2-log decrease in CFU’s in the stainless steel group. This did not reach the 5-log decrease necessary for a treatment to classify as bactericidal [28] (as seen in some antibiotic treatments [29]). Nevertheless, the observed 2-log decrease in bacterial growth is encouraging and justifies further in-vivo studies to evaluate these in-vitro results [30]. The in-vivo reaction to implants depends on a variety of factors (i.e. trauma and immunocompromised patients are more prone to infection compared to otherwise healthy patients with a closed fracture) [2]. As such, future in-vivo work will require evaluation to test the performance of thermal cycling in reducing the formation of colony forming units under multiple scenarios.

## Conclusions

Overall, these in-vitro findings show there is potential for thermal cycling with respect to decreasing the number of colony forming units on orthopaedic stainless steel and titanium implants. If bacterial load is lowered and shows a reduction in clinical implant infections, it would have enormous implications in the treatment of orthopaedic trauma, deformity and degenerative conditions.

## Abbreviations

CaCl_2_: Calcium chloride
CFU: colony forming unit
DCP: dynamic compression plate
FMNH2: Flavin mononucleotide
IQR: interquartile range
LB: lysogeny broth
MgCl_2_: Magnesium chloride
PBS: phosphate buffered saline
*S.aureus*: *Staphylococcus aureus*
TSBG: tryptic soy broth with glucose
